# The balance of Bmp6 and Wnt10b regulates the telogen-anagen transition of hair follicles

**DOI:** 10.1186/s12964-019-0330-x

**Published:** 2019-02-21

**Authors:** Pan Wu, Yiming Zhang, Yizhan Xing, Wei Xu, Haiying Guo, Fang Deng, Xiaogen Ma, Yuhong Li

**Affiliations:** 1 Department of Cell Biology, Army Medical University, Gaotanyan street No. 30, Shapingba, Chongqing, 400038 China; 2 Department of Plastic and Cosmetic Surgery, Xinqiao Hospital, Army Medical University, Chongqing, China; 3 Department of Dermatology, Chongqing First People’s Hospital and Chongqing Traditional Chinese Medicine Hospital, Chongqing, China

**Keywords:** Hair follicle regeneration, Wnt10b, BMP6, Hair follicle stem cells

## Abstract

**Background:**

The periodic growth of hair follicles is regulated by the balance of activators and inhibitors. The BMP signaling pathway plays an important role during hair follicle regeneration, but the exact BMP protein that controls this process has not been revealed.

**Methods:**

The expression of BMP6 was determined via in situ *hybridization* and immunofluorescence. The in vivo effect of BMP6 overexpression was studied by using a previously established adenovirus injection model. The hair follicle regeneration was assessed by gross observation, H&E staining and 5-bromo-2-deoxyuridine (BrdU) tracing. The expression patterns of BMP6 signaling and Wnt10b signaling in both AdBMP6-treated and AdWnt10b-treated skins were determined by in situ *hybridization* and immunofluorescence.

**Results:**

BMP6 was expressed differently in the stages of hair follicle cycle. The telogen-anagen transition of hair follicles was inhibited by adenovirus-mediated overexpression of BMP6. In the in vivo model, the BMP6 signaling was inhibited by Wnt10b and the Wnt10b signaling was inhibited by BMP6. The activation of hair follicle stem cells (HFSCs) was also competitively regulated by Wnt10b and BMP6.

**Conclusions:**

Combined with previously reported data of Wnt10b, our findings indicate that BMP6 and Wnt10b are major inhibitors and activators respectively and their balance regulates the telogen-anagen transition of hair follicles. To the best of our knowledge, our data provide previously unreported insights into the regulation of hair follicle cycling and provide new clues for the diagnosis and therapies of hair loss.

**Electronic supplementary material:**

The online version of this article (10.1186/s12964-019-0330-x) contains supplementary material, which is available to authorized users.

## Background

The hair follicle is a specific mini-organ with the ability to undergo periodic growth and is an effective model for tissue regeneration research. Usually, postnatal follicles have three growth stages: anagen, catagen and telogen. Research on the regulation of the hair follicle cycle is focused on anagen onset. Previous reports showed that in late telogen, the balance between activators and inhibitors may determine whether a hair follicle can reenter anagen [1–3]. Activators and inhibitors may be of intrafollicular or extrafollicular origin [4]. Many signaling pathways, such as the Wnt (Wingless-type mouse mammary tumor virus integration site), BMP (Bone morphogenetic protein), FGF (Fibroblast growth factor) and Shh (Sonic hedgehog) pathways, are involved in the anagen onset of the hair follicle. These signaling pathways play important roles in the development and growth of many organs, including hair follicles. However, the dominant signaling pathway is unknown.

Wnt3a and Wnt10b are secreted glycoproteins reported to transduce signals through the canonical Wnt/β-catenin signaling pathway, while some other ligand such as Wnt5a, utilize other signaling pathways such as the Wnt/PCP (planar cell polarity) pathway and the Wnt/Ca^2+^ signaling pathway. BMP proteins are also secreted proteins. When the BMP protein binds with its receptor BMPR, the Smad1/5/8 complex will be phosphorylated and combined with Smad4. The Smad1/5/8/4 complex will be translocated to the nucleus and regulate the expression of target genes. In the regulation of the homeostasis and cyclic activation of hair follicle stem cells (HFSCs), the competitive balance of intrabulge Wnt and BMP signaling plays important roles [5]. However, the exact Wnt or BMP proteins involved in the regulation of the hair follicle cycle have not been revealed. Wnt1 and BMP2 were reported to be the two factors involved in recruiting multipotent neural crest progenitors isolated from adult bone marrow [6]. In the regulation of the hair follicle cycle, the specific Wnt/BMP proteins may be different. Previously, we reported that Wnt10b is a major activator in hair follicle regeneration and it promoted the growth of hair follicles via Wnt/β-catenin signaling pathway [7, 8]. Based on published RNA-seq data [9], we compared the expression data between dermal papilla cells and dermal fibroblasts in the postnatal day 5 mouse skin. Among all the *bmp* genes, *bmp6* had the highest relative expression in dermal papilla cells and was expressed in the outer root sheath. In this study, we identified BMP6 as an important inhibitor in hair follicle regeneration. In addition, we showed that Wnt10b inhibited the BMP signaling pathway and BMP6 inhibited the Wnt signaling pathway. To our knowledge, our data provide previously unreported evidence demonstrating that the balance of Wnt10b and BMP6 regulates hair follicle regeneration.

## Methods

### Animals and vectors

Male C57 BL/6 mice were obtained from the Laboratory Animal Center of the Army Medical University, Chongqing, China. All animal-related procedures were conducted in strict accordance with the approved institutional animal care and maintenance protocols. All experimental protocols were approved by the Laboratory Animal Welfare and Ethics Committee of the Third Military Medical University. Adenovirus vectors were constructed and propagated as described previously [10]. AdBMP6 and AdWnt10b are similar to AdGFP but contain the coding region of mouse BMP6 and Wnt10b, respectively.

### In vivo injection of adenovirus

For the injection of AdBMP6, C57 BL/6 mice at postnatal day 56 (telogen) were anesthetized with 1% pentobarbital sodium. Back hairs were depilated with a blend of resin and beeswax and 25 μL of adenovirus vector was injected intradermally along the median dorsal line of the skin. The injection was angled toward the head and produced a 1-cm wheal. A circle drawn by a cotton bud dipped with picric acid labeled the wheal. The skin and hair follicles of the mice were observed every day. Mice were sacrificed to observe the inner structure and protein changes at 1, 2, 3, 7, 10 and 14 days post-injection. Mice at postnatal day 30 (anagen onset) were also used for the injection of AdBMP6, but the depilation step was omitted. For the injection of AdWnt10b, C57 BL/6 mice at postnatal day 56 (telogen) were used and the depilation step was also omitted. To label the proliferating cells, 100 μL BrdU (Sigma, USA) was injected intraperitoneally at 4 h before sacrifice. To label the proliferated cells, 100 μL BrdU was injected intraperitoneally at the same time as the adenovirus treatment. BrdU was prepared with 10 mg/mL in 0.9% sodium chloride.

### H&E staining

Dorsal skins were fixed in 4% paraformaldehyde, gradually dehydrated through a graded series of alcohol, embedded in paraffin and cut into 5-μm-thick sections. After gradual hydration, sections were stained with hematoxylin (Zhongshan Goldenbridge, China) for 2 min and subsequently rinsed with water. The sections were later stained with eosin (Zhongshan Goldenbridge, China) for 2 min and rinsed with water thereafter. After gradual dehydration, the sections were mounted with neutral gum (Zhongshan Goldenbridge, China) and observed under a microscope.

### Immunofluorescence

Samples were embedded in paraffin and cut into 5-μm-thick sections. The primary antibodies against the following proteins were used: Sox4, Krt10, invulcrin, AE13 (all diluted 1:100, Santa Cruz, USA), Krt14 (1:50, Sangon Biotech, China), Krt15 (1:50, Sangon Biotech, China), AE15 (1:100, Abcam, USA), BrdU (1:200, Abcam, USA), BMP6 (1:100, Abcam, USA) and Wnt10b (1:100, Abcam, USA). Cy3-labeled or AF488-labeled secondary antibodies (Beyotime, China) were used. The sections were counterstained with DAPI (Beyotime, China). Finally, the sections were mounted with anti-fade mount media (Beyotime, China) and observed under a microscope.

### In situ hybridization

Digoxin-labeled probes were synthesized according to the manufacturer’s instructions (Dig RNA labeling kit, Roche). Before hybridization, slides were dewaxed and hydrated under RNase-free conditions, digested in 10 μg/mL proteinase K, refixed with fresh 4% paraformaldehyde and prehybridized in prehybridization solution (in a volume of 50 ml, add formamide 25 mL, 20 × SSC 12.5 mL, Tween-20 50 μL, 10% Chaps 500 μL, 10 mg/ml tRNA 1 mL, 500 mM EDTA 500 μL, 10 mg/ml Heparin 250 μL, Blocking Reagent 1 g, DEPC H_2_O to 50 mL). Slides were incubated overnight with BMP6 probes (diluted to 100 ng/mL with prehybridization solution) at 65 °C. After hybridization, the slides were washed with 2 × SSC, 0.2 × SSC and PBT, preblocked with 20% goat serum in PBT for 2 h; and incubated overnight with the anti-DIG-AP antibody (diluted 1:1000, Roche) at 4 °C. The slides were then washed with NTMT (in a volume of 50 ml, 2.5 ml 2 M Tris-HCl pH 9.5, 1.25 ml 2 M MgCl_2_, 1 ml 5 M NaCl, 12 mg Levamisole and 50 μl Tween-20 were added) and stained with BM purple (Roche) according to the manufacturer’s instructions. When the color had developed, the slides were washed with PBS. Finally, the slides were mounted with neutral gum.

### Statistical analysis

For the animal experiments, 6 male C57 BL/6 mice were used for each group. For each phase of the hair cycle, at least 6 images were acquired, which include at least 20 follicles. Six hair follicles were randomly selected for each group to perform statistical analysis. Student’s T-test was used to compare the ratio of positive cells. *P* < 0.05 was considered as significantly different.

## Results

### BMP6 expression is associated with the hair cycle

**Fig. 1:**
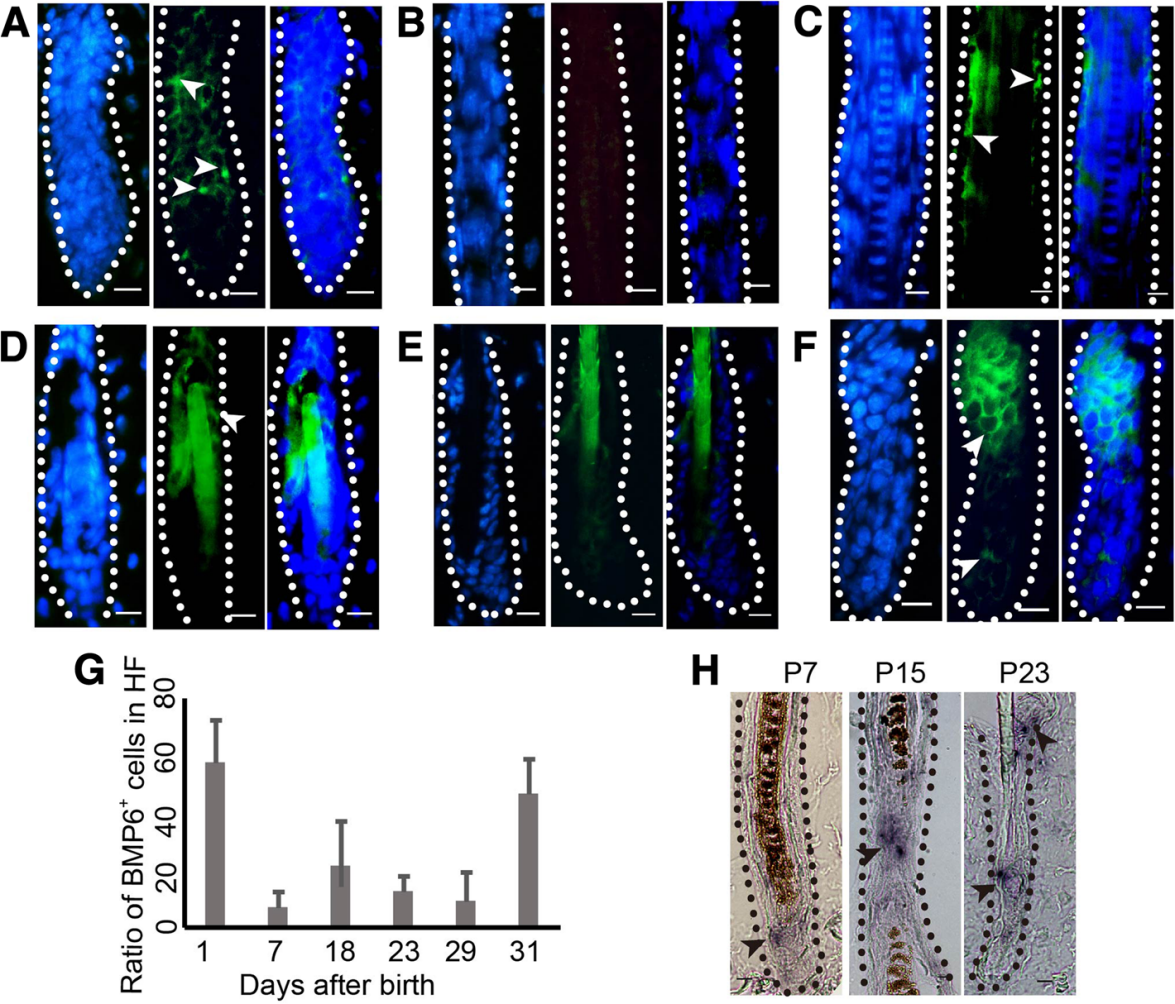
The expression of BMP6 in hair follicle cycle. **a**-**f** The protein expression of BMP6 in hair follicle cycle. From left to right: DAPI, BMP6, Merge of DAPI and BMP6. The dashed lines depict the structure of hair follicles. **a** P11, (**b**) P7, (**c**) P18, (**d**) P23, (**e**) P29, (**f**) P31. **g** The bar chart of the protein expression of BMP6 in hair follicle cycle as showing in (**a**-**f**). *N* = 6. **h** The mRNA expression of BMP6 in hair cycle. Arrowheads show the positive expression of BMP6. HF, hair follicle. Scale bar = 10 μm

BMP6 was expressed in all stages of the hair cycle (Fig. 1). The expression structures include the outer root sheath (Fig. 1a, c, and d), bulge (Fig. 1f, upper part) and matrix (Fig. 1 f, lower part). However, the expression levels were different. Specifically, compared with those in middle anagen (Fig. 1b, h-P7), late anagen (Fig. 1h-P15), catagen (Fig. 1c) and telogen (Fig. 1d, h-P23), the ratio of BMP6 positive cells was much higher in early anagen (Fig. 1a, f, and g). This finding suggests that BMP6 may play a role in the hair cycle, especially in early anagen.

### BMP6 inhibits the telogen-anagen transition in vivo

**Fig. 2:**
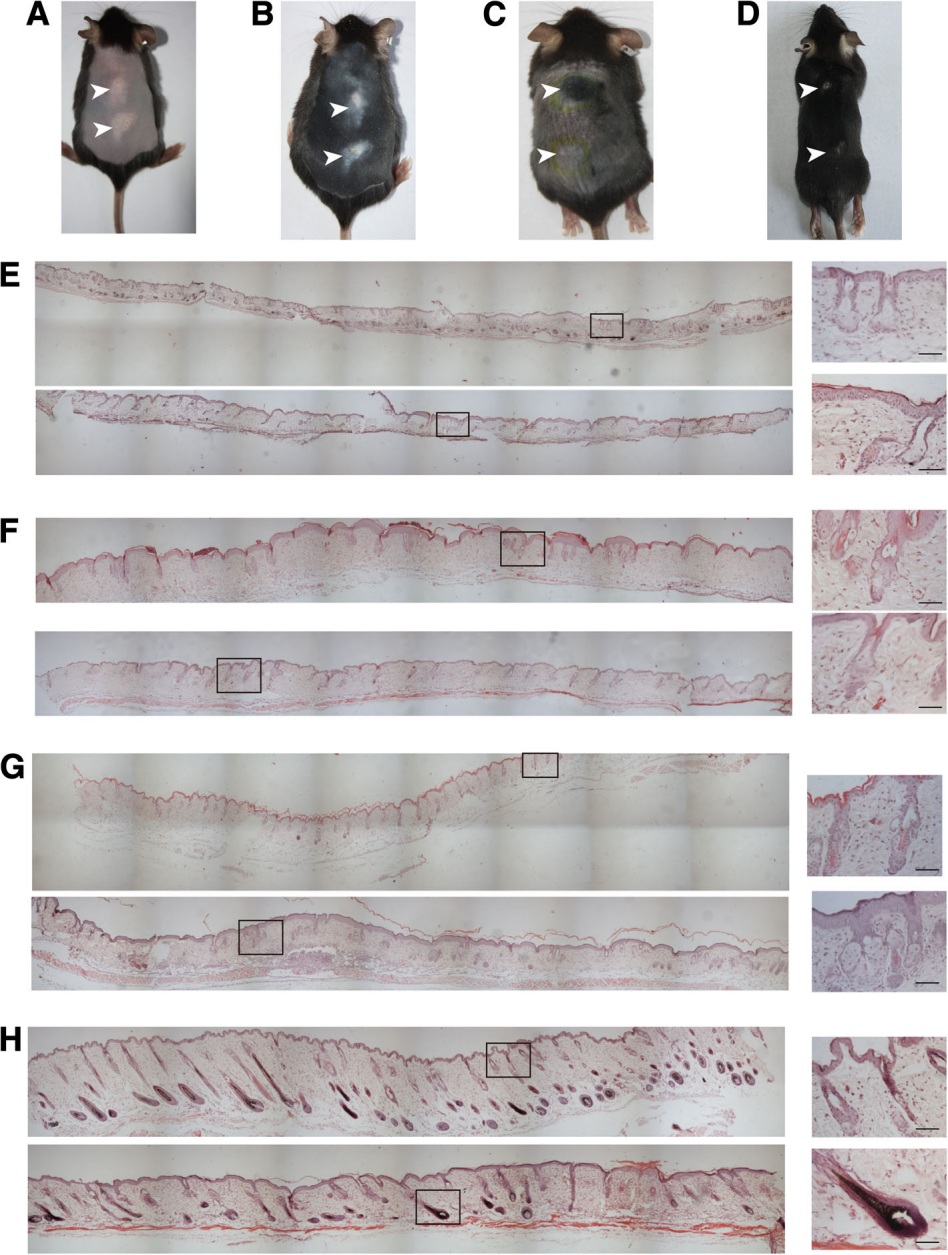
BMP6 inhibits the transition of telogen to anagen. **a**-**b** Six days (**a**) and 10 days (**b**) after the injection of AdBMP6 into the skin of depilated telogen C57 mice. **c** Thirteen days after the injection of AdNoggin (the site near the head) or AdGFP (the site near the tail) into the shaved skin of telogen C57 mice. **d** Nine days after the injection of AdBMP6 into the C57 mice at anagen onset. The arrowheads show the injection sites. **e**-**f** H&E staining of skin samples injected with AdBMP6 or AdGFP. The injection sites were sampled at 1 day after treatment (**e**), 2 days after treatment (**f**), 3 days after treatment (**g**) and 7 days after treatment (**h**). For each section, the upper part is the AdBMP6 injected sample, while the lower part is the AdGFP injected sample. The right panels are the enlarged pictures of the framed area in the left panels. Scale bar = 50 μm

To determine the role of BMP6 in the hair cycle, we introduced a synchronization model of hair follicles and injected AdBMP6 intradermally into the skin of synchronized C57 mice. After AdBMP6 injection, BMP6 was expressed in the hair follicles of the injected area (Additional file 1: Figure S1). At 6 days after injection, the hair follicles in the AdBMP6-injected area of 14:18 (77.8%) mice were still in telogen, whereas the hair follicles in the non-injected area or AdGFP-injected area of 18:18 (100%) mice had already entered anagen (Fig. 2a). This phenomenon was enhanced at 10 days after injection when the hair stem of the hair follicles in the non-injected area or AdGFP-injected area had already grown out of the skin and the skin in the non-injected area changed to black (Fig. 2b). HE staining demonstrated that the differences between the AdBMP6-injected area and AdGFP injected area appeared at an earlier time. At 24 h after injection and 48 h after injection, the hair follicles between the two groups did not show obvious differences (Fig. 2e, f, Additional file 1: Figure S2A, B). At 72 h after injection, the hair follicles in the AdGFP injected area entered anagen, whereas the hair follicles in the AdBMP6-injected area remained in telogen (Fig. 2g, Additional file 1: Figure S2C). At 7 days after injection, the hair follicles in the AdBMP6-injected area also entered anagen, but were at a relatively immature stage (Fig. 2h, Additional file 1: Figure S2D).

We also injected AdBMP6 into the skin of normal anagen onset C57 mice. This treatment inhibited the anagen onset of the hair follicles in 4:6 (66.7%) mice (Fig. 2d). Noggin is a classical inhibitor of BMP signaling. When AdNoggin was injected into the full telogen skin of C57 mice, hair follicles grew out at 13 days after injection, while the hair follicles in the non-injected area or AdGFP injected area were still in telogen (Fig. 2c). These findings further indicate that BMP6 may inhibit the telogen-anagen transition of the hair cycle.

### BMP6 inhibits the activation of HFSCs

**Fig. 3:**
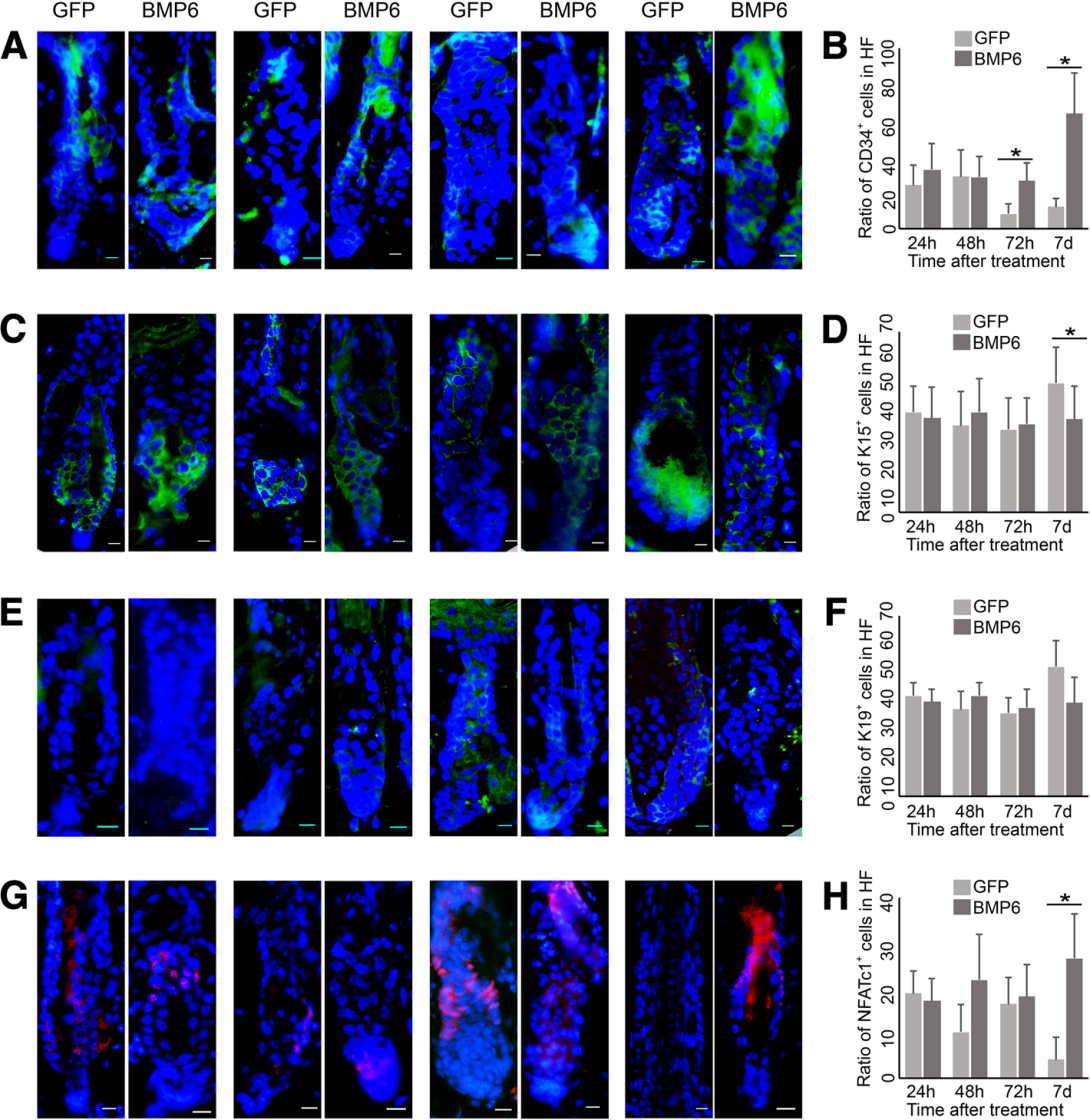
Expression pattern of HFSC markers in the AdBMP6 treated hair follicles. The hairs of telogen C57 mice were depilated and the skin was injected with AdBMP6 or AdGFP intradermally. The expression pattern of HFSC markers CD34 (**a**, **b**), Krt15 (**c**, **d**), Krt19 (**e**, **f**) and NFATc1 (**g**, **h**) was determined by immunofluorescence. The nuclei were counterstained by DAPI. **a**, **c**, **e**, **g** From left to right: 1 day after treatment, 2 days after treatment, 3 days after treatment and 7 days after treatment. Scale bar = 10 μm. **b**, **d**, **f**, **h** The bar charts of the ratio of positive cells as showing in (**a**, **c**, **e**, **g**). The ratio was normalized by the number of cells in hair follicle. HF, hair follicle. *N* = 6, *P < 0.05

In normal hair follicles, BMP6 was expressed in the bulge area. To determine the role of BMP6 in HFSCs, we analyzed HFSCs in AdBMP6-treated hair follicles at 24 h after injection, 48 h after injection, 72 h after injection and 7 days after injection. The number of CD34-positive cells was higher in the AdBMP6-treated group at 72 h after injection and 7 days after injection (Fig. 3a, b). The number of Krt15-positive cells was lower in the AdBMP6-treated group at 7 days after injection (Fig. 3c, d). The Krt19 expression did not show an obvious difference between AdBMP6-treated group and AdGFP-treated group (Fig. 3e, f). The number of NFATc1 positive cells was higher in the AdBMP6-treated group than the AdGFP-treated group at 7 days after injection (Fig. 3g, h). These data demonstrate that BMP6 inhibits the activation of HFSCs.

### BMP6 and Wnt10b competitively regulate telogen-anagen transition in the hair cycle

**Fig. 4:**
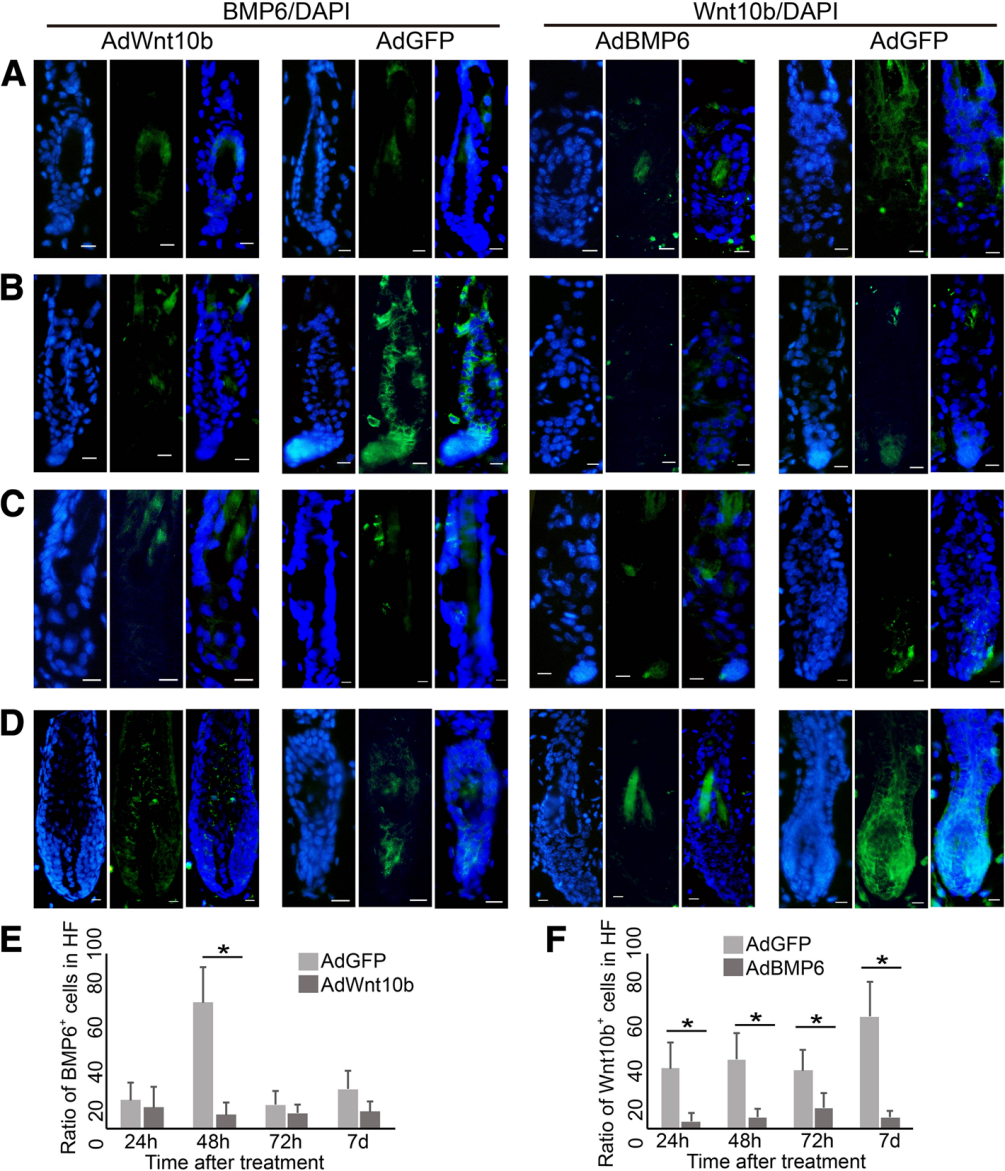
Expression pattern of Wnt10b in AdBMP6 treated and BMP6 in AdWnt10b treated hair follicles. Left two panels: AdWnt10b or AdGFP was injected into the telogen back skin of C57 mice. Right two panels: AdBMP6 or AdGFP was injected into the depilation-induced anagen onset back skin of C57 mice. The expression of BMP6 or Wnt10b was determined by immunofluorescence at 1 day after treatment (**a**), 2 days after treatment (**b**), 3 days after treatment (**c**) and 7 days after treatment (**d**). DAPI was used to counterstain the nucleus. Scale bar = 10 μm. (**e**, **f**) The bar charts of the ratio of positive cells as showing in (**a**-**d**). The ratio was normalized by the number of cells in hair follicle. HF, hair follicle. *N* = 6, *P < 0.05

Because Wnt10b signaling also plays a dominant role in the telogen-anagen transition of hair follicles, we analyzed the expression of Wnt10b in AdBMP6- or AdGFP- treated anagen onset skin. From 24 h after injection to 7 days after injection, Wnt10b was rarely expressed in the AdBMP6-treated group (Fig. 4a-d, right). The ratio of Wnt10b positive cells in hair follicle was always higher in the AdGFP-treated group than in the AdBMP6-treated group (Fig. 4f). At 48 h after injection, Wnt10b was expressed in the hair germ area (Fig. 4c, right). At 7 days after injection, Wnt10b was expressed in the outer root sheath, hair matrix and precortex area (Fig. 4d, right). We also analyzed the expression of BMP6 in AdWnt10b treated telogen skin. The ratio of BMP6 positive cells in hair follicle did not show an obvious difference at 24 h or 72 h after injection. However, At 48 h and 7 days after injection, the ratio of BMP6 positive cells in hair follicle was higher in the AdGFP-treated group than in the AdWnt10b-treated group (Fig. 4a-d, left. e).

### BMP6 inhibits the proliferation of HFSCs

**Fig. 5:**
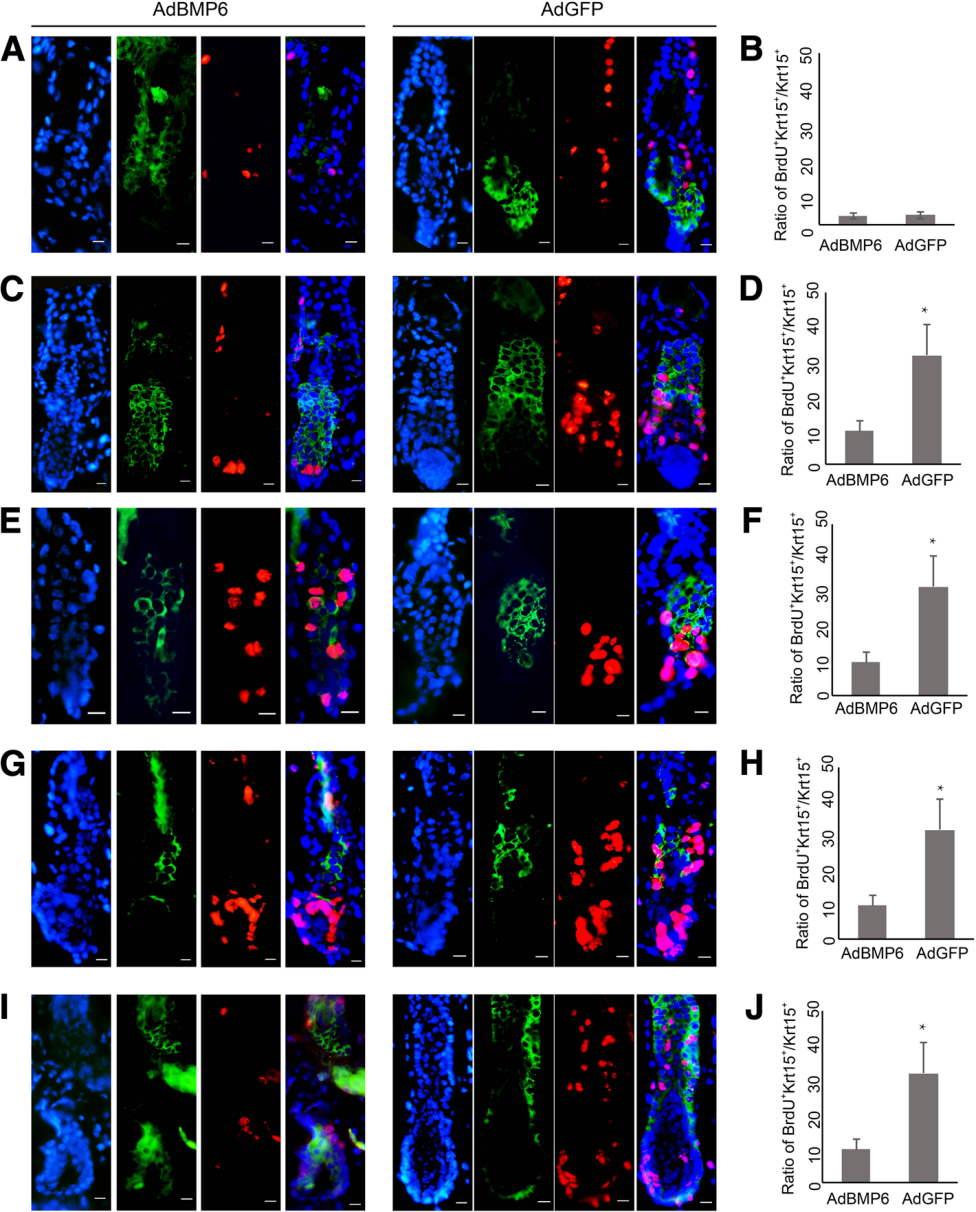
BrdU labeling in KRT15 positive HFSCs. Krt15 and BrdU were detected by immunofluorescence at 1 day after treatment (**a**, **b**), 2 days after treatment (**c**, **d**), 3 days after treatment (**e**-**h**) and 7 days after treatment (**i**, **j**). **a**-**f**, **i**, **j** BrdU was injected at 4 h before sampling. **g**, **h** BrdU was injected at the time of adenovirus treating. Green color shows the positive expression of Krt15. Red color shows the BrdU positive cells. DAPI was used to counterstain the nucleus. **a**, **c**, **e**, **g**, **i** The right panels are the merged picture of protein markers and DAPI. Scale bar = 10 μm. **b**, **d**, **f**, **h**, **j** are the bar charts of the ratio of BrdU positive cells in Krt15 positive cells. HF, hair follicle. *N* = 6, * *P* < 0.05

Previously, we reported that overexpression of Wnt10b activated HFSCs in vivo [11]. We analyzed the proliferation status of HFSCs by 5-bromo-2-deoxyuridine (BrdU) labeling after the overexpression of BMP6. After 4 h of tracing, the incorporation of BrdU was detected in both AdBMP6-treated and AdGFP-treated hair follicles. The detected locus included the outer root sheath, matrix and bulge. Since 24 h after the injection, the number of BrdU-positive cells was lower in the AdBMP6-treated group (Fig. 5a, c, e, g, i). The incorporation of BrdU in Krt15 positive cells indicates proliferating HFSCs. The incorporation ratio of BrdU in Krt15 positive cells was also lower in the AdBMP6-treated group than the AdGFP-treated group since 48 h after treatment (Fig. 5b, d, f, h, and j).

## Discussion

As a major subgroup of the transforming growth factor β superfamily, BMPs are essential morphogens that participate in tissue patterning. BMPs also function in tissue regeneration and adult stem cell differentiation. For example, BMP signaling inhibits the differentiation and migration of mesenchymal stem cells [12]. BMP signaling also functions in hair regeneration and HFSC activation [13]. BMP signaling is required for HFSC quiescence and to promote transit amplifying cell differentiation along different lineages as the hair cycle progresses [14]. However, previous studies of BMPs have mostly focused on BMP2, BMP4 and BMP9 [15]. Some BMPs may have opposite functions [16]. Although BMP6 has been studied in several organs, little research on in hair follicles has been performed [17–19]. In cancer, the status of Smad4 and P53 determines the effect of BMP signaling on Wnt signaling. This molecule can act as either an enhancer or inhibitor [20]. We sought to determine the role of BMP6 during hair follicle regeneration. Hair follicles enter anagen immediately after the depilation of the old telogen hair stems. The telogen-anagen transition of hair follicles was inhibited when AdBMP6 was injected intradermally into the induced skin. Noggin is reported to be an inhibitor of BMP signaling and is essential for tissue development [21, 22]. The telogen hair follicles entered anagen when AdNoggin was injected intradermally into the skin. These data prove that BMP6 is a key inhibitor of telogen-anagen transition.

Usually, several signaling pathways act together during the development and regeneration of tissues [23]. Previously, we reported that Wnt10b is a key activator of telogen-anagen transition. Here, we found that BMP6 was a key inhibitor of telogen-anagen transition. The Wnt signaling pathway and BMP signaling pathway have been reported to act together in tissue development and stem cell differentiation. However, the effects are not always the same. On one hand, the Wnt/β-catenin signaling pathway and BMP signaling pathway can be synchronically regulated [24, 25], can synergistically act to maintain neural crest stem cells and iPS cells [26, 27] and can synergistically act to regulate the osteogenic differentiation of mesenchymal progenitors [28]. On the other hand, the Wnt/β-catenin signaling pathway and BMP signaling pathway can act competitively in intestinal epithelial growth and stem cell self-renew [29, 30]. Wnts can also suppress lateral cell fates by antagonizing Bmp4 expression [31].

Regarding the regeneration of hair follicles, sequential inhibition and activation of BMPR1a are necessary to define a band of hair progenitor cells, which possess Wnt signaling activation [32, 33]. BMP signaling represses the activation of HFSCs [2]. The Wnt signaling pathway and BMP signaling pathway may be competitive in regulating HFSC activation [5]. BMP6 has already been reported to be an inhibitor for hair follicle regeneration [34, 35]. As we reported here and previously, Wnt10b is a key activator and BMP6 is a key inhibitorfor HFSC behavior control. However, the crosstalk relationship between the two signaling pathways during the hair regeneration cycle has not been comprehensively investigated previouysly. After AdWnt10b treatment, the expression of BMP6 remained low in telogen hair follicles. In the control group, the expression of BMP6 increased at 48 h after treatment and decreased to a low level at 72 h after treatment (Fig. 4, left). This finding suggests that adenovirus-mediated overexpression of Wnt10b suppresses the endogenous expression of BMP6. After AdBMP6 treatment, the expression of Wnt10b remained low in telogen hair follicles. In the control group, Wnt10b expression increased as the hair follicles entered anagen (Fig. 4, right). These results suggest that adenovirus-mediated overexpression of BMP6 suppresses the expression of endogenous Wnt10b. These data indicate that Wnt10b and BMP6 function competitively in hair follicle regeneration.

**Fig. 6:**
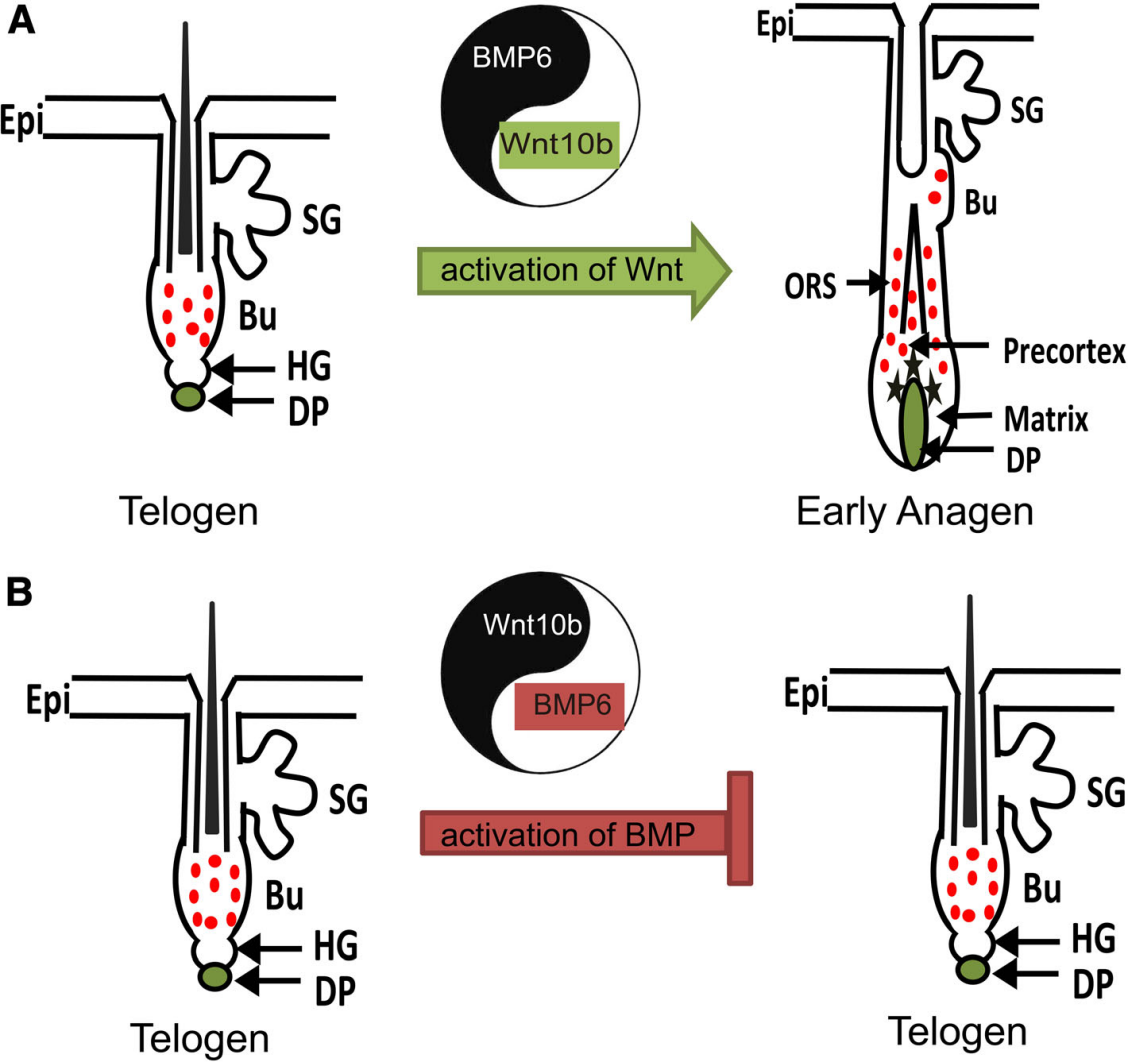
Yin-Yang model of hair follicle regeneration tuned by Wnt/BMP signaling. The balance of BMP6 and Wnt10b regulates the telogen-anagen transition of hair follicles. **a** When Wnt10b is in the dominant role, it activates the Wnt signaling pathway and inhibits BMP signaling pathway, thus activates the HFSCs. Along with the proliferation of HFSCs, hair follicles enter anagen. **b** When BMP6 is in the dominant role, it activates the BMP signaling pathway and inhibits Wnt signaling pathway, thus inhibits the activation of HFSCs. HFSCs keep quiescent and hair follicles stay in telogen. Epi, epithelia. SG, sebaceous gland. Bu, bulge. HG, hair germ. DP, dermal papilla. ORS, outer root sheath. The red dots indicate HFSCs express Krt15

Several proteins were reported to be HFSC markers. They are all expressed in the bulge area. However, the expression patterns of the markers are not always the same. To systematically assess the HFSCs, we determined the expression of CD34, Krt15, Krt19 and NFATc1 in AdBMP6-treated hair follicles. Overall, the quiescent marker NFATc1 had higher expression levels in the AdBMP6-treated group, whereas the relatively active marker Krt15 had a lower expression level in AdBMP6 treated group. The expressions of Krt19 were not obviously different (Fig. 3). In addition, proliferating cells labeled with BrdU in hair follicle were less abundant in the AdBMP6-treated group than in the AdGFP-treated group (Fig. 5). These data demonstrate that BMP6 is an inhibitor of HFSC activation. On the other hand, we previously reported that Wnt10b was an activator of HFSC activation [11]. These findings combined with the in vivo results, indicate that BMP6 and Wnt10b competitively regulate the activation of HFSCs. Based on previously reported data and our findings, we propose a Yin-Yang model for the regulation of the telogen-anagen transition in hair follicles. The dominant role of BMP6 or Wnt10b activates the BMP signaling pathway or Wnt signaling pathway, thus inhibiting or stimulating the activation of HFSCs, which leads to the telogen-anagen transition of hair follicles (Fig. 6). However, high expression of both BMP6 and Wnt10b was observed during early anagen. Thus other factors may be involved in upregulating BMP6 expression during early anagen. These factors remain to be uncovered in future research.

## Conclusions

In summary, we report the inhibitory effect of BMP6 during the telogen-anagen transition of hair follicles. This molecule can independently inhibit the activation of HFSCs. We also report that BMP6 inhibits the expression of Wnt10b and that Wnt10b inhibits the expression of BMP6. We conclude that BMP6 and Wnt10b function competitively to regulate the activation of HFSCs and the telogen-anagen transition of hair follicles. This work identified two key factors that regulate the activation of HFSCs and hair follicle regeneration. To the best of our knowledge, this is the first report that discusses the relationship between Wnt10b and BMP6. However, the conclusions are based on adenovirus-mediated overexpression, these findings should be confirmed in transgenic animals and may be used to promote the development of therapeutic drugs for dysfunctional hair follicle regeneration.

## Additional file

Additional file 1: **Figure S1.** Expression of BMP6 in AdBMP6 treated hair follicle. **Figure S2.** Expression pattern of hair structure markers in AdBMP6 treated hair follicle (PDF 1846 kb)

## Abbreviations

AdBMP6: Adenovirus with bone morphogenetic protein 6
AdGFP: Adenovirus with green fluorescent protein
AdNoggin: Adenovirus with Noggin
AdWnt10b: Adenovirus with Wnt10b
BMP: Bone morphogenetic protein
Wnt: Wingless-type mouse mammary tumor virus integration site
