# Correction to: The balance of Bmp6 and Wnt10b regulates the telogen-anagen transition of hair follicles

**DOI:** 10.1186/s12964-020-0508-2

**Published:** 2020-01-07

**Authors:** Pan Wu, Yiming Zhang, Yizhan Xing, Wei Xu, Haiying Guo, Fang Deng, Xiaogen Ma, Yuhong Li

**Affiliations:** 1 Department of Cell Biology, Army Medical University, Gaotanyan street No. 30, Shapingba, 400038 Chongqing China; 2 Department of Plastic and Cosmetic Surgery, Xinqiao Hospital, Army Medical University, Chongqing, China; 3 Department of Dermatology, Chongqing First People’s Hospital and Chongqing Traditional Chinese Medicine Hospital, Chongqing, China

**Correction to: Cell Commun Signal (2019) 17:16**

**https://doi.org/10.1186/s12964-019-0330-x**

Following publication of the original article [1], the authors reported that they would like to correct the second last sentence of “Authors’ information” section as PW is an undergraduate, but was incorrectly described as a Ph.D. in the sentence. The sentence should read “PW is an undergraduate. YZ, YX, WX, HG, FD and YL are Ph.D.”. The authors sincerely apologize for having this unintentional error in the article, and apologize for any inconvenience caused.
