# Supplementary material for: The balance of Bmp6 and Wnt10b regulates the telogen-anagen transition of hair follicles
Source: Cell Commun Signal. 2019 Feb 21;17:16. doi: 10.1186/s12964-019-0330-x (PMC6385416; doi:10.1186/s12964-019-0330-x)
Supplement: Supplementary file 1 — Figure S1. Expression of BMP6 in AdBMP6 treated hair follicle. Figure S2. Expression pattern of hair structure markers in AdBMP6 treated hair follicle (PDF 1846 kb) [file 12964_2019_330_MOESM1_ESM.pdf]

Supplementary Figures

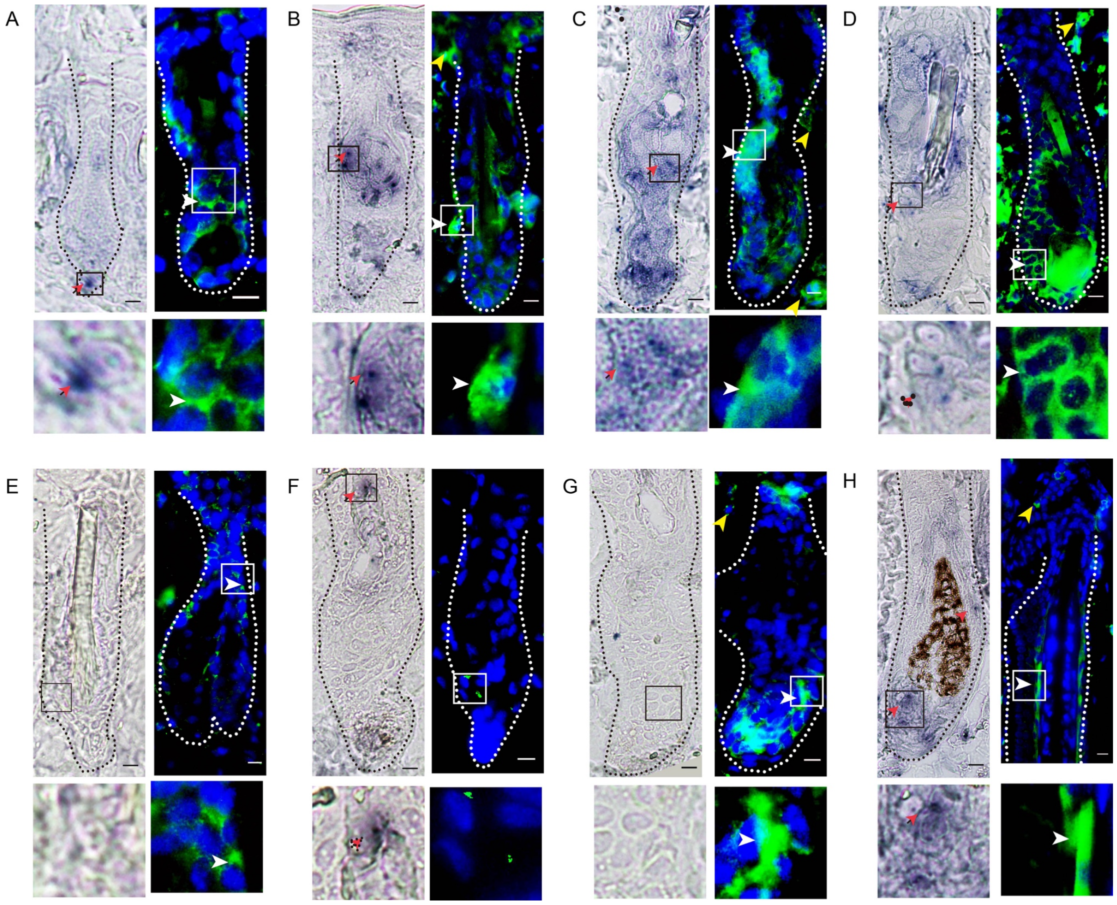

Figure S1 Expression of BMP6 in AdBMP6 treated hair follicle

The hairs of telogen C57 mice were depilated and the skin were injected with AdBMP6 (A-D) or AdGFP (E-H) intradermally. The expression patterns of BMP6 were determined by in situ hybridization and immunofluorescence at 1 day after treatment (A, E), 2 days after treatment (B, F), 3 days after treatment (C, G) and 7 days after treatment (D, H). For each labeled small picture, the left panel shows the *in situ* hybridization result, while the right panel shows the immunofluorescence result. The lower part of each panel shows the enlarged picture of the framed area in the upper part. Dashed line depicts the structure of hair follicle. Arrowheads show the positive expression of BMP6. Scale bar=10 μm.

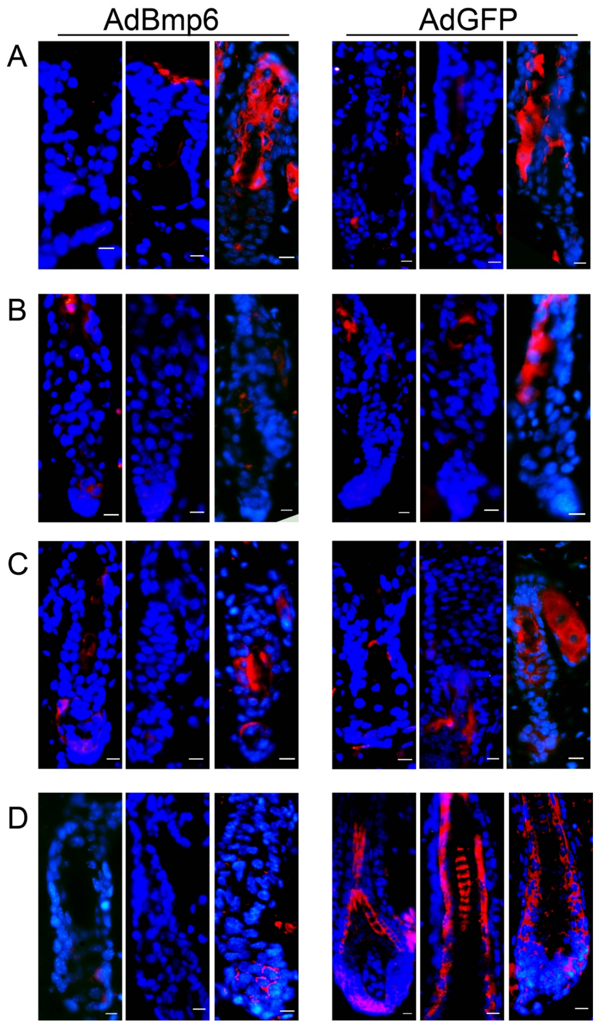

Figure S2 Expression pattern of hair structure markers in AdBMP6 treated hair follicle

The hairs of telogen C57 mice were depilated and the skin were injected with AdBMP6 or AdGFP intradermally. The expression patterns of AE13, AE15 and MSX2 were determined by immunofluorescence at 1 day after treatment (A), 2 days after treatment (B), 3 days after treatment (C) and 7 days after treatment (D). For the left 3 panels: AE13, AE15 and MSX2 expression in AdBMP6 treated samples. For the right 3 panels: AE13, AE15 and MSX2 expression in AdGFP treated samples. Scale bar=10 μm.
